# Physiologically Adaptive Soft Millirobot for Atraumatic, Image-Guided Endovascular Therapy

**DOI:** 10.21203/rs.3.rs-8089047/v1

**Published:** 2025-11-19

**Authors:** Tuan Anh Le, Husnu H. Alabay, Prabh G. Singh, Melissa Gioia Austin, Pamela A. Manco Urbina, Sanjay Misra, Hakan Ceylan

**Affiliations:** 1 Department of Physiology and Biomedical Engineering, Mayo Clinic, Scottsdale, Arizona.; 2 Division of Vascular and Interventional Radiology, Mayo Clinic, Rochester, Minnesota.; 3 Labor and Delivery, Abrazo Health, Glendale, Arizona.

## Abstract

Atraumatic and localized drug delivery to the vascular endothelium remains a critical unmet need in interventional medicine, with major implications for the management of arterial and venous diseases. Current approaches, such as drug-eluting stents and drug-coated balloons, combine mechanical revascularization with local drug release but frequently denude the endothelium and injure vessel walls, leading to restenosis, thrombosis, and chronic inflammatory repair cycles that severely limit long-term outcomes. Here, we present EndoBot, a soft, untethered millirobot designed for atraumatic navigation and targeted endovascular drug delivery during physiologic blood flow. EndoBot employs magnetically actuated corkscrew propulsion and mechanically adaptive surface crawling to maintain low radial pressure (<1 kPa) and preserve endothelial integrity. Its feasibility is established in arterial and venous phantom models, ex vivo human umbilical veins under normothermic perfusion, and in vivo rat inferior vena cava, all under clinical fluoroscopic guidance using a human-scale magnetic manipulation system. Despite periodic vessel motion, compression, and geometric irregularities, EndoBot maintains stable, mechanically adaptive navigation without endothelial injury. Under real-time fluoroscopic imaging, we identify key challenges and showcase clinically feasible strategies for 3D localization and tracking, overcoming intrinsic depth limitations of 2D clinical imaging. The device can be deployed and retrieved through standard vascular sheaths and remains stable under supraphysiologic arterial and venous flow conditions (flow rate >100 mL min^−1^; flow velocity >155 cm s^−1^shear stress >10 Pa).Blood-compatibility testing demonstrates no increase in coagulation tendency and minimal hemolysis (<0.01%) during magnetic actuation. For drug delivery, EndoBot employs a novel endoluminal painting-based technique that transfers a hydrophobic and flow-resistant coating directly onto the inner vessel surface. This method ensures uniform deposition of a biodegradable drug depot without generating fragments larger than typical capillary diameters (>10 mm). By enabling local, atraumatic drug delivery without compromising vessel wall integrity, EndoBot first time demonstrates a translationally viable platform for exploring effectiveness in preventive interventions in early-stage vascular disease and for adjunctive therapies in advanced disease stages.

## INTRODUCTION

Vascular diseases represent a substantial global health challenge, accounting for over 18 million deaths annually and placing a substantial burden on health care systems worldwide^1–3^. Current treatments for advanced vascular occlusions primarily involve balloon angioplasty and stents, pairing mechanical revascularization with localized pharmacologic delivery, performed on millions of patients each year^4^. Despite their initial success, these procedures frequently lead to complications, such as restenosis and thrombosis, which can compromise their clinical effectiveness^5^. Central to these complications is the excessive mechanical force applied during balloon angioplasty and stenting procedures. Typical balloon contact pressures (20–100 kPa), depending on to the compliance of the balloon and vascular tissue, both substantially exceed the physiologic pressure tolerance of endothelial cells (ECs) (approximately 12.4 kPa) lining the vessel lumen^6–11^. This mechanical trauma disrupts the ECs, injures the vessel wall, and triggers inflammatory processes that drive neointimal growth and thrombosis^12^.

ECs have a pivotal role in regulating immune responses, maintaining vascular hemostasis, preventing thrombosis, and inhibiting smooth muscle cell proliferation^13–17^. To mitigate the damage, localized delivery of antiproliferative drugs to the vessel wall through drug-coated balloons (DCBs) or drug-eluting stents (DESs) has been actively explored^18–20^. DCBs, however, continue to pose risks of EC injury, even though they were specifically designed to reduce mechanical trauma through lower inflation pressures (approximately 600–800 kPa). Antiproliferative drugs can further delay endothelial repair, exacerbating risks of restenosis, thrombosis, and long-term vascular dysfunction^21,22^. Consequently, patients often need repeated vascular interventions, which perpetuate injury/recovery cycles, progressive restenosis, and increased arterial stiffness, as well as escalate the risk of vessel rupture with each subsequent treatment. To counter thrombosis, prolonged dual antiplatelet therapy (typically 6–12 months) is routinely prescribed after endovascular procedures. This practice substantially elevates bleeding risks and can hinder the natural healing process at the site of vascular injury^21,23^.

Given the pivotal role of ECs in maintaining vascular function, the endothelium represents a clinically significant therapeutic target, particularly during the early stages of vascular disease^24^. Nevertheless, despite decades of innovation, no targeted modality exists that can safely deliver drugs or genetic engineering machinery directly onto a discrete segment of the vascular endothelium while preserving its integrity^25^. Although nanocarrier-based approaches have been explored for targeted deposition, challenges related to precise spatial localization and systemic toxicity have often been encountered^26^. Consequently, a major unmet need remains for a new class of atraumatic tools capable of endothelial-safe, localized drug delivery to the luminal surface of an intact vasculature without physical disruption.

Addressing this need can lead to primary preventive intervention strategies that target early and middle stages of vascular disease progression. Rather than the current focus on late-stage revascularization and preventing restenosis, primary preventive approaches could stop or even reverse the progression of occlusive disease at an early stage. However, the lack of completely atraumatic intervention methods has prohibited exploring these promising early treatments effectively.

Untethered microrobots have recently emerged as a promising route toward minimally invasive, site-specific intervention^27^. Magnetic actuation offers remote, wireless control with deep tissue penetration, eliminating the need for on-board power or tethers. However, most magnetic micro- and millirobotic systems have been demonstrated only in simplified environments such as transparent microfluidic channels or static phantoms^28–38^. Translation to clinical settings remains hindered by several key challenges: (i) robust locomotion within a human-scale workspace, (ii) mechanical adaptability to dynamic blood flow and vessel motion, (iii) stability under supraphysiologic flow, (iv) compatibility with medical imaging systems (v) atraumatic vessel wall interaction under physiologic flow, (vi) blood compatibility, (vii) wireless deployment and retrieval, (viii) controlled endoluminal drug transfer. Overcoming these barriers is essential for achieving clinically viable, image-guided microrobotic therapy.

Here, we introduced a physiologically compatible, untethered, soft and mechanically adaptive millirobot (EndoBot), designed for precise navigation and localized endoluminal payload delivery within blood vessels ranging from 1 to 4 mm in diameter (Table S1). This clinically relevant scale spans small to midsized arteries and veins, including, peripheral arteries, and superficial veins, coronary and intracranial branches.

**Figure 1.**
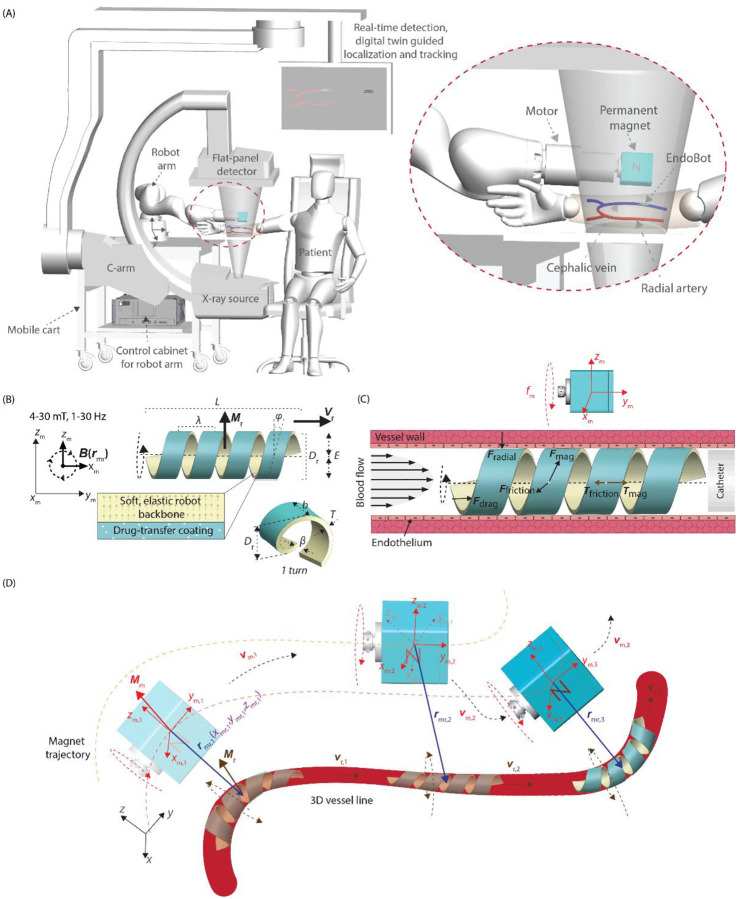
Clinical vision for EndoBot: Concept of an untethered soft millirobot for atraumatic endovascular drug delivery. **(A)** A human-scale magnetic manipulation system integrates a robot-arm-mounted rotating permanent magnet with real-time fluoroscopy based, virtual reality enhanced detection, localization, and control for atraumatic, image-guided vascular intervention. Such an approach could enable preventive or immunomodulatory local therapies on the vessel walls, for example to maintain patency of arteriovenous fistulas in hemodialysis patients. **(B)** Design of the physiologically adaptive EndoBot. EndoBot consists of a hollow, elastic backbone with a flat outer surface engineered to maximize luminal contact while maintaining low wall stress. The hydrophobic, flow-resistant lubricant coating forms a uniform drug transfer interface for leak-minimized, local delivery. **(C, D)** Mechanically adaptive locomotion under physiologic flow. The refined thin-walled helical architecture provides high radial and circumferential deformability, allowing stable surface crawling and corkscrew propulsion without disturbing bulk blood flow. During operation, the robot arm dynamically adjusts the magnet’s orientation and position to generate propulsive torque vectors aligned with EndoBot’s instantaneous pose under fluoroscopy. This active steering enables precise 3D navigation through tortuous or deforming vessels, while EndoBot’s compliant body passively adapts to curvature and compression for safe, resilient, atraumatic wall interaction and localized drug delivery.

EndoBot operates through rotating magnetic field actuation under real-time fluoroscopic guidance within a human-scale workspace, establishing the feasibility of a practical framework for image-guided, atraumatic endovascular therapy (Fig. 1A). This work represents the first in vivo demonstration of a fully untethered, magnetically guided, mechanically adaptive endovascular device functioning safely. By enabling precise navigation and localized payload delivery directly onto the endothelium, EndoBot addresses a critical unmet need in developing next-generation robotic interventions, which are entirely non-invasive. Such approaches could ultimately support preventive or immunomodulatory therapies targeting localized vascular inflammation, for example in maintaining arteriovenous fistula (AVF) patency in hemodialysis patients, where recurrent endothelial injury remains a major challenge.

EndoBot features 2 main components: a soft elastic robot backbone and a lubricant-based hydrophobic drug-transfer coating. The backbone is formed as a thin, magnetic, hollow helix, drawing inspiration from biologic locomotors, such as bacterial flagella^39^, self-burying plant seeds^40^, and miniature robot designs proposed for swimming^32,35,41–44^ (Fig. 1B). Besides, EndoBot structure maximizes luminal contact while maintaining low wall stress, high recovery after radial load, luminal patency and sufficient flexibility for safe deformation within tortuous vessels.

Adaptive surface crawling is central to EndoBot’s motion (Fig. 1C). The hollow helical structure allows bulk blood flow to pass through its core while maintaining stable contact with the vessel wall. This configuration resists displacement by drag forces and enables propulsion both with and against the flow. Frictional coupling between the outer surface and endothelium provides traction for controlled movement and direct drug transfer under physiologic flow conditions.

The thin-walled, elastic architecture confers radial and circumferential compliance, allowing EndoBot to conform to vessel curvature, non-uniform cross-sections, and dynamic motions such as pulsation or vasospasm (Fig. 1D). During navigation, a robot-arm-mounted rotating magnet continuously adjusts its orientation and position to align the magnetic torque with EndoBot’s instantaneous pose, generating active propulsion while the soft body passively accommodates local geometry. As EndoBot traverses the lumen, its lubricant coating, loaded with a flow-resistant therapeutic compound, is gently transferred onto the endothelial surface, forming a uniform, biodegradable drug depot without fragmenting to compromise the perfusion. Through this synergy of active magnetic control and passive mechanical adaptation, EndoBot achieves stable, atraumatic navigation and sustained, localized drug delivery under clinically realistic flow and imaging conditions.

In this study, we established the feasibility of the EndoBot drug-delivery platform in normothermically perfused phantom vessels, ex vivo human umbilical veins, and in vivo rat inferior vena cava (IVC) considering human-scale operational constraints and within established safety limits. Extensive performance tests and comprehensive design of the platform provide a strong basis for its translational potential. Future efforts will focus on validating EndoBot and evaluating its capabilities in both small and large animal models particularly for preventive interventions targeting vascular endothelial inflammation and vessel walls, along with clinically relevant follow-up times. Ultimately, EndoBot could improve patient outcomes by shifting the paradigm in vascular disease management from reactive treatments to proactive and preventive strategies against intimal hyperplasia and stenosis.

## RESULTS

### Design, Fabrication, and Refinement of EndoBot

#### Helical Design for Endovascular Drug Delivery

A helical soft robot design for endoluminal locomotion offers substantial safety and operational advantages over conventional designs such as tubular or stent-like structures. Unlike soft-bodied tubes, which may deform unpredictably under dynamic vascular compression, the helical form can inherently conform to nonuniform vessel geometries with minimal stress on the surrounding endothelium. When exposed to external forces from pulsatile flow, turbulence, or vessel-wall interactions, soft-bodied tubes can develop localized bulges or kinks. Such protrusions may unintentionally project into the vessel lumen, disturbing the bulk flow, increasing drag, and introducing flow separation or turbulence. These deformations not only compromise navigational stability but also elevate the risk of embolization or clot formation.

In contrast, the helical topology of EndoBot constrains cross-sectional deformation along a predefined spatial path, minimizing protrusions and ensuring more predictable flow-compatible behavior under dynamic vascular conditions. This geometric adaptability enables the robot to traverse sharply curved and nonuniform anatomic paths with reduced risk of damaging the vessel wall. Moreover, helical configuration enables robust, torque-driven corkscrew locomotion, which efficiently converts rotational input into axial displacement, allowing the robot to self-propel even under high-shear flow conditions. The distributed contact of the helix with the vessel surface improves traction and directional control during movement, further enhancing navigation robustness.

Although magnetically reprogrammable beam-to-helix transformations have previously enabled twisted soft robots to navigate fluid-filled channels, such designs often suffer from excessive bending freedom, compromising directional control and mechanical stability^35^. Asymmetric forces due to pulsatile blood flow, turbulence, and vasoconstriction can result in dynamic and unpredictable changes in vessel geometry. These fluctuations challenge the responsiveness of real-time, magnetic field–based actuation, complicating reliable control and elevating the risk of erratic motion or device-induced embolization.

To address these challenges, EndoBot was fabricated with a helical structure from the outset and with carefully tuned mechanical properties that restricted its degrees of freedom and allowed adaptive deformation only within predefined radial boundaries and curvatures—across its cross-section, along its main helical axis, and along curved paths. These structural constraints enhanced mechanical predictability without sacrificing necessary compliance. The tailored helical geometry, combined with elastic material composition, has equipped EndoBot with mechanical shape-memory behavior, enabling dynamic recovery from both permissible and unexpected deformations. Collectively, these refinements substantially enhanced the operational safety and reliability of EndoBot.

#### Fabrication

We established a high-fidelity, soft lithographic fabrication method to systematically refine the structural and compositional features of EndoBot for nominal robot diameters ($D_{\text{r}}$) ranging from 1.0 to 4.0 mm (Supplementary text 1, Figs. S1-S5). The EndoBot backbone was fabricated from a polydimethylsiloxane (PDMS)-based composite (Sylgard 184, Dow Inc) embedded with magnetic neodymium, iron, and boron (NdFeB) powder (D50, 5 mm, Magquench GmbH). This magnetic elastomer composite is highly reproducible and offers tailorable elasticity and magnetic responsiveness, ensuring robust performance under various operational conditions. Additionally, the industrial-scale availability of these materials aligns with US Food and Drug Administration (FDA) requirements for consistent, scalable manufacturing and regulatory compliance, bolstering translational feasibility.

$D_{\text{r}}$ was selected based on the nonuniform lumen profile of the vessel, ensuring that EndoBot accommodates diameter variations along the targeted segment. Additional helical parameters, including length (L), helical pitch length (λ), helical blade thickness (b), groove size (β), and thickness (T) are scaled relative to $D_{\text{r}}$ using constant coefficients (Table S2). Helical angle (φ) was not scaled to $D_{\text{r}}$ and remained constant. These relationships were established through rational design requirements, empirical measurements for performance refinement, and earlier studies of optimized corkscrew microswimmers designed for low Reynolds-number environments (Supplementary text 2).

Although the scaling coefficients originate from the nominal, as-fabricated dimensions, some parameters ($D_{\text{r}}$, L, and λ) dynamically adjust relative to one another under vascular confinement and magnetic propulsion. For example, maintaining an $L/D_{\text{r}}$ ratio greater than 3.0 enables more efficient application of magnetic torque along the major helical axis, reducing the risk of tumbling under off-axis loads^32,45,46^. Therefore, EndoBot was constructed with a nominal $L/D_{\text{r}}$ ratio of 3.3, which can dynamically range from 3.3 to 7.0 during navigation due to partial elongation in the axial dimension and compression in the radial dimension. This trend of adaptability helps sustain effective torque application and robust corkscrew propulsion, even in narrower vessel segments where elevated friction and drag forces could otherwise impede locomotion.

The L/λ ratio, which represents the number of helical turns, also governs the capacity of the helix to behave like a mechanical spring under radial compression. In its as-fabricated state, EndoBot has an L/λ ratio of 4.8, which can increase up to 6.7 when navigating smaller vessels than $D_{\text{r}}$. Such adaptability allows the structure to distribute stress along its length, storing elastic energy without forming localized high-strain regions that might otherwise cause buckling or intrusion into the vessel wall. By creating new conformal turns under compression, the helix preserves its structural integrity, enabling smooth navigation while ensuring uninterrupted blood flow and vessel wall safety.

#### Refinements

Thickness (T) was identified as a key variable for tuning the deformation behavior of the helix. By optimizing T, a high mechanical cost can be imposed on erratic deformations while still allowing permissible deformation during motion (Supplementary text 2). Although physiologic radial loads for EndoBot are circumferential, in assessing the radial force of the EndoBot, flat-plate compression was used as a practical proxy. As validated in Fig. S6 and Supplementary text 2, this method yields comparable force measurements to circumferential compression, supporting its use for characterizing radial force.

The effective elastic modulus (E) across the cross-section scales nearly cubically with $T\left(E\propto T^{3.05}\right)$, creating a substantial energy barrier to stochastic bending with even moderate increases in T (Fig. S7). Moreover, increasing T substantially enhances the postcompression recovery of the EndoBot, which is a critical factor for sustained surface crawling during navigation (Fig. S8).

However, higher T also increases both the intrinsic ($F_{\text{intrinsic}}$) and extrinsic ($F_{\text{extrinsic}}$) components of radial force:

$$F_{\text{radial}}=F_{\text{intrinsic}}+F_{\text{extrinsic}}$$

where $F_{\text{intrinsic}}$ arises from the stored elastic energy under compressive strain $\left(\left|F_{\text{intrinsic}}\right|\propto T^{3.05}\right)$, and $F_{\text{extrinsic}}$ is created by the magnetic pulling force $\left(\left|F_{\text{extrinsic}}\right|\propto T\right)$ (Fig. S7). At the same time, although a larger T improves the stability, it reduces both the conformal deformation capacity under magnetic actuation and vascular patency (Figs. S9, S10, S11). Consequently, T was judiciously optimized to balance robust structural integrity and mechanical compliance for adaptive crawling while minimizing radial pressure on the vessel wall and preserving patency. Finally, we refined the NdFeB mass ratio within the PDMS matrix and adjusted curing of the elastomer to further tune E and maximize magnetic responsiveness without compromising vascular patency (Fig. S7, Table S3).

For drug-transfer efficiency, the contact area between EndoBot and the vessel wall was maximized by employing a high b/β ratio while minimizing φ (Fig. S12). A smaller φ not only increases wall contact but also reduces the cross-sectional area occupied by EndoBot, thereby improving overall vessel patency (Fig. S12).

Building on these design priorities and comprehensive refinements, we established a mechanically scalable blueprint for showcasing in diverse vascular and flow conditions (Supplementary text 3, Tables S2, S4, Figs. S13, S14).

### Establishing Operational Limits for Endovascular Navigation Performance

**Figure 2.**
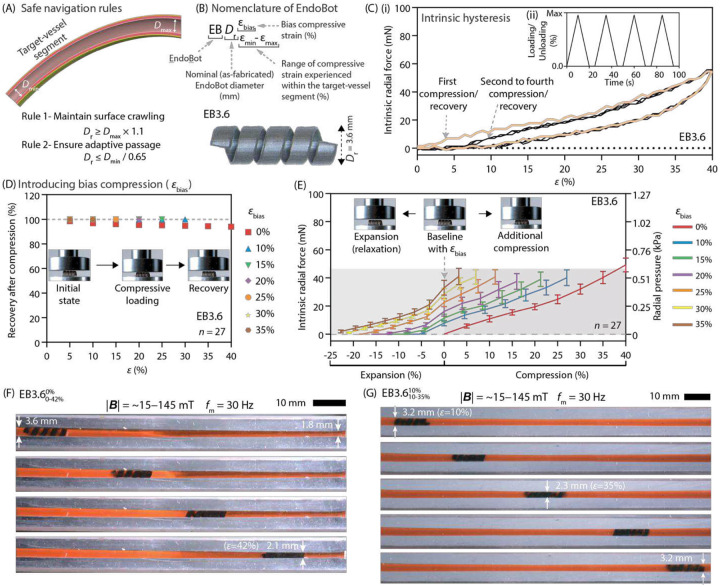
Establishing safe navigation limits for EndoBot. **(A)** EndoBot selection rules for a given vascular segment characterized with a minimum ($D_{\text{min}}$) and maximum ($D_{\text{max}}$) vessel diameters. Two key rules govern the appropriate selection of EndoBot for a given target vessel. Rule 1: EndoBot must maintain a minimum bias compressive strain of 10%, ensuring its effective diameter ($D_{\text{r}}$) is at least $1.1\times D_{\text{max.}}$. Rule 2: EndoBot must operate under a maximum compressive strain of 35%, meaning $D_{\text{r}}$ should not exceed 65% of $D_{\text{min}}$. **(B)** Nomenclature and key operational features of EndoBot. EB represents EndoBot and $D_{\text{r}}$ (in mm) is its as-fabricated diameter. The superscript $\varepsilon_{\text{bias}}$ indicates the bias compressive strain planned for applying based on the angiography-estimated minimum vessel diameter, whereas the subscript $\varepsilon_{\text{min}}-\varepsilon_{\text{max}}$ specifies the range of compressive strain experienced during navigation under magnetic actuation. **(C)** Intrinsic radial force ($F_{\text{intrinsic}}$) under cyclic deformation. **(i)** Measurement of the intrinsic radial force generated by EB3.6 when subjected to cyclic compressive strain up to 40%. **(ii)** Time-course profile of the cyclic deformation pattern. **(D)** Diameter recovery to biased diameter ($D_{\text{bias}}$) after loading/unloading cycles. **(E)** Intrinsic radial force ($F_{\text{intrinsic}}$) and radial pressure of EB3.6 with bias compressive strain during compression and recovery. **(F)** Conformal deformation capacity measured in a tapered vessel. ${\text{EB}3.6}_{0\%-42\%}^{0\%}$ is shown under a rotating magnetic field in a blood-filled, conical phantom vessel with diameters tapering from 3.6 mm to 1.8 mm. **(G)** Adaptive surface crawling in a nonuniform vessel. Testing of ${\text{EB}3.6}_{10\%-35\%}^{10\%}$ in a phantom vessel with diameters ranging from 3.2 mm to 2.3 mm under a rotating magnetic field, confirming safe and adaptive crawling locomotion within its operational safety limits.

To effectively deploy EndoBot in a target vessel, it is critical to evaluate the minimum ($D_{\text{min}}$) and maximum ($D_{\text{max}}$) luminal diameters of the vessel segment. For stable surface crawling, a $D_{\text{r}}$ must be selected that ensures continuous contact with the vessel wall. This selection should also accommodate variations in vessel diameter through dynamic radial compression and expansion (Fig. 2A). When testing an EndoBot prototype with a nominal diameter of 3.6 mm (hereafter referred to as EB3.6 according to the nomenclature in Fig. 2B), it was subjected to cyclic compressive deformation from 0% to 40% strain (ε). During this testing, a mild hysteresis effect was observed. After the initial loading-unloading cycle, the device recovered approximately 94% of its original diameter, stabilizing at this slightly reduced dimension in subsequent cycles (Fig. 2C, Supplementary movie 1). To mitigate hysteresis, a bias compressive strain ($\varepsilon_{\text{bias}}$) was introduced, thus preloading the EndoBot with a minimum strain that was maintained throughout navigation. Practically, $\varepsilon_{\text{bias}}$ was implemented by choosing $D_{\text{r}}$ larger than $D_{\text{max}}$, such that:

$$D_{\text{bias}}=D_{\text{r}}-\left(D_{\text{r}}\times \left(\frac{\varepsilon_{\text{bias}}}{100}\right)\right)$$

This strategy offsets the initial elastic energy loss and ensures complete recovery to the biased diameter ($D_{\text{bias}}$) corresponding to the maximum lumen diameter. We learned that applying a minimum $\varepsilon_{\text{bias}}$ of 10% ensures complete diameter recovery to $D_{\text{bias}}$ (Figs. 2D, S15). Accordingly, we defined *Operational Limit Rule 1:* EndoBot must maintain at least 10% bias compressive strain ($\varepsilon_{\text{bias}}\geq 10\%$) along the target vessel. Therefore, to sustain effective surface crawling:


$$D_{\text{r}}\geq D_{\text{max}}\times 1.1$$

Increasing $\varepsilon_{\text{bias}}$ beyond the minimum requirement can store additional elastic energy upon device deployment and further reduce elastic energy loss (Table S5). This additionally stored energy enables EndoBot to expand and maintain wall contact when encountering larger vessel cross-sections during navigation (Fig. 2E). For example, at $\varepsilon_{\text{bias}}=30\%$, EB3.6 safely recovers around 24% diameter variation, providing a robust margin to maintain contact across varying vessel diameters. Importantly, even at maximum compression (ε=40%), the radial pressure exerted by EndoBot remains below 0.75 kPa , which is an order of magnitude below the endothelial damage threshold (approximately 12.4 kPa), thus suggesting atraumatic mechanical interaction with vessel walls^9,10^.

Next, we evaluated the safe conformal deformation capacity of EndoBot by deploying EB3.6 within a blood-filled conical phantom vessel tapering from 3.6 mm to 1.8 mm (Fig. 2F, Supplementary text 4, Table S6, Fig. S16). Although a 50% diameter variation exceeds typical clinical conditions in early or moderate-stage occlusive disease, this approach provides a single, continuous model to systematically test the upper deformation limits of EndoBot. EB3.6 successfully navigated the phantom vessel until approximately 42% lumen narrowing (Supplementary movie 1). Beyond this point, magnetic torque could not overcome combined resistive effects arising from increasing stored elastic energy ($U_{\text{elastic}}\propto \varepsilon^{2}$) and amplified radial force and frictional resistance ($\left|F_{\text{radial}}\right|\propto \varepsilon$ and $\left|F_{\text{friction}}\right|\propto \left|F_{\text{radial}}\right|$). As a result, to ensure consistent, safe navigation and avoid vessel blockage, an additional 7% safety margin was introduced, establishing *Operational Limit Rule 2:*

$$\varepsilon_{\text{max}}\leq 35\%,\text{or}\;\text{equivalently},D_{\text{r}}\leq \frac{D_{\text{min}}}{0.65}$$

This conservative margin ensures robustness of the robot and uninterrupted blood flow, although a higher coefficient of friction on the surface of the phantom vessels than on real blood vessels suggests greater deformation within a live vessel (Fig. S17, Supplementary text 5).

Combining Rules 1 and 2 provides practical guidance. For example, for a target vessel segment with $D_{\text{max}}=3.2\;\text{mm}$ and $D_{\text{min}}=2.3\;\text{mm}$, selecting $D_{\text{r}}=3.6\;\text{mm}$ satisfies both criteria, allowing EB3.6 to navigate safely without risking excessive deformation or losing surface contact. Demonstration using EB3.6 in a phantom vessel within this diameter range confirmed adaptive and secure crawling locomotion (Fig. 2G, Supplementary movie 1).

### Stability and Propulsion of EndoBot During Physiologic and Supraphysiologic Blood Flow

#### Static and Kinetic Stability

A safe EndoBot design must remain stationary without drifting and must avoid obstructing blood flow at physiologic velocities typically encountered in small- to medium-sized arteries and veins with lumen diameters ranging from 1.0 to 3.0 mm (Table S1). This stability is primarily achieved by balancing static friction at the robot-lumen interface with the drag forces exerted by blood flow at the robot’s surface (Supplementary text 5, Figs. S15, S16). To better recapitulate the physiologic hemodynamic variabilities of veins and arteries, we assessed experiments using both a peristaltic pump and a pulsatile pump emulating human cardiac output.

**Figure 3.**
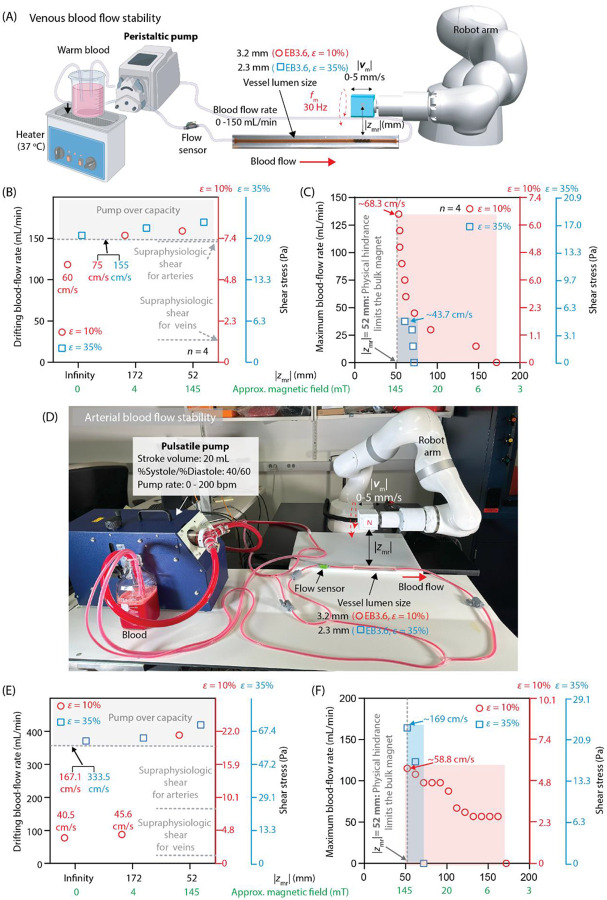
Stability of EndoBot under venous and arterial blood flow. **(A, D)** Experimental setups and flow conditions with peristaltic pump providing steady flow and with pulsatile pump emulating human cardiac output to mimic venous and arterial flow, respectively. (**B**, **E**) Static stability. Maximum blood flow rate and blood-flow velocity endured by ${\text{EB}3.6}_{10\%}^{10\%}$ and ${\text{EB}3.6}_{35\%}^{35\%}$ before drifting with the blood flow. The shaded region denotes flow rates exceeding the operational range of the pump. (**C**, **F**) Kinetic stability during locomotion. Maximum blood-flow rates permitting safe navigation of ${\text{EB}3.6}_{10\%}^{10\%}$ and ${\text{EB}3.6}_{35\%}^{35\%}$ with or against the blood flow under the minimum external magnetic field required for propulsion.

For static stability, static frictional forces must exceed the drag forces to prevent the EndoBot from drifting (Supplementary text 6, Figs. S18, S19). To evaluate these dynamics, we measured the maximum blood-flow rates and velocities that caused EB3.6 to drift in phantom vessels under the minimum and maximum allowable compressive strains $\left(\varepsilon_{\text{min}}=10\%,\varepsilon_{\text{max}}=35\%\right)$ (Fig. 3, Supplementary text 7, Fig. S20).

Both conditions demonstrated remarkable stability. Under peristaltic blood flow, EB3.6 tolerated flow rates up to 120 mL/min (60 cm/s) and 150 mL/min (155 cm/s) at ε=10% and 35%, respectively. In both setups, this performance was achieved without the assistance of external magnetic fields (Figs. 3B, 3E). When an external magnetic field as low as 4 mT was applied, stability under both peristaltic and pulsatile blood flow increased due to the appearance of extrinsic friction the appearance of $F_{\text{extrinsic}}$ friction caused by the magnetic pulling force $F_{\text{mag}}$ (Supplementary texts 2, 8, Figs. S21-S25). Under peristaltic blood flow, the static stability exceeded the maximum flow-rate capacity of the pump (150 mL/min) at both ε=10% and 35%. This flow rates correspond to physiologic conditions observed in arteries and veins such as the middle cerebral artery, coronary arteries, brachial artery, radial vein, and cephalic vein. Human blood flow during anesthesia in these vessels typically ranges between 5 and 150 mL/min, depending on vessel size and hemodynamic state (Table S1). Under pulsatile blood flow, static stability was up to 87 mL/min at ε=10% and exceeded the maximum flow-rate capacity of the pump (358 mL/min) at ε=35%. These values are supraphysiologic for the given vessel sizes, further highlighting robust device stability.

The wall shear stress experienced by the inner surface of EB3.6 within these phantom vessels ranged from approximately 5 to 25 Pa ^47^, substantially surpassing the physiologic shear stress values typically observed in human vessels of comparable diameter (2.4–3.3 mm): 0.1–0.6 Pa in veins and 1–7 Pa in arteries (Supplementary text 7, Fig. S20). Notably, these elevated shear stress values closely align with the high-shear hemodynamic conditions observed clinically in arteriovenous fistulas (AVFs) of dialysis patients, especially at the arterial anastomosis (4.2–10 Pa) and the venous segment near the anastomosis (1.3–5.1 Pa)^48^.

To assess stability under more challenging conditions, we introduced random pinches and folds into the tube a few centimeters upstream from the robot to create irregular and turbulent flow patterns. Even under these dynamic and disturbed flow conditions, EndoBot maintained stable positioning without drift or displacement, demonstrating robust anchoring capability. Such resilient performance under extreme flow conditions highlights EndoBot’s potential suitability for demanding endovascular applications, including deployment within dynamic, high-flow and high-shear vascular environments, such as those observed in clinical AVFs.

To further assess EndoBot stability under kinetic friction, we rotated it at a constant frequency of 30 Hz by applying a magnetic field on EB3.6 up to 145 mT. We then determined the maximum flow rates at which it could still be reliably propelled in the direction of flow and against flow under the minimum external magnetic field required for motion. While 35% compressive strain demonstrated superior stability under static friction conditions for both peristaltic and pulsatile blood driven flow regimes, kinetic stability showed different trends. 10% strain resulted in more effective kinetic stability against higher flow rates (Fig. 3C); whereas, under pulsatile flow, 35% strain provided greater kinetic stability (Fig. 3F).

Specifically, under peristaltic flow while ${\text{EB}3.6}_{10\%}^{10\%}$ maintained kinetic stability up to a flow rate of 135 mL/min (68.3 cm/s), ${\text{EB}3.6}_{35\%}^{35\%}$ was limited to a flow rate of 38 mL/min (43.7 cm/s). This disparity under peristaltic flow was primarily attributed to the friction forces and their changing roles under static and kinetic conditions (Fig. S16). Static stability strongly depends on the frictional force magnitude relative to drag. At ε=10%, the frictional force is relatively low and can be overcome by the drag force, making the EB3.6 susceptible to losing static stability with increasing flow rates. At ε=35%, the significantly larger frictional force far exceeds the drag force, ensuring robust static stability even at higher flow rates. To achieve kinetic stability, however, the propulsive force must overcome the sum of the frictional force and the drag force. Consequently, the significantly lower resistance at ε=10% preserves a larger fraction of the available propulsive force, enabling ${\text{EB}3.6}_{10\%}^{10\%}$ to sustain controlled locomotion against substantially higher flow rates.

In contrast, under pulsatile flow, kinetic stability was maintained with ${\text{EB}3.6}_{10\%}^{10\%}$ up to a flow rate of 115 mL/min (58.8 cm/s), a slight decrease compared to the peristaltic flow performance. The behavioral shift was due to the unique hemodynamic profile of the system. The systolic phase—typically lasted longer with the pulsatile flow than in it was in peristaltic flow (Fig. S20), generating higher peak flow rates and shear forces, increasing resistance to upstream motion.

However, the striking shift of behavior was observed with ${\text{EB}3.6}_{35\%}^{35\%}$ where the kinetic stability was substantially higher, up to 164 mL/min (169 cm/s). In this case, we observed that the EndoBot experienced a transient reduction in effective compression due to cyclic vessel expansion observed only under high pulsatile flow conditions (Supplementary Movie 2). This temporary expansion dynamically reduces dynamic friction, enabling ${\text{EB}3.6}_{35\%}^{35\%}$ to maintain motion at higher flow rates despite its otherwise high frictional resistance. This effect does not lead to similarly pronounced impact for the EndoBot with ε=10%, where the friction is already sufficiently low in both flow types, so the effect of vessel expansion is negligible.

Therefore, we concluded that while low bias strain (10%) is advantageous under low pulsation conditions, such as veins, high bias strain (35%) can become more favorable for locomotion within arteries due to frictional compensation from vessel compliance.

#### Propulsion

**Figure 4.**
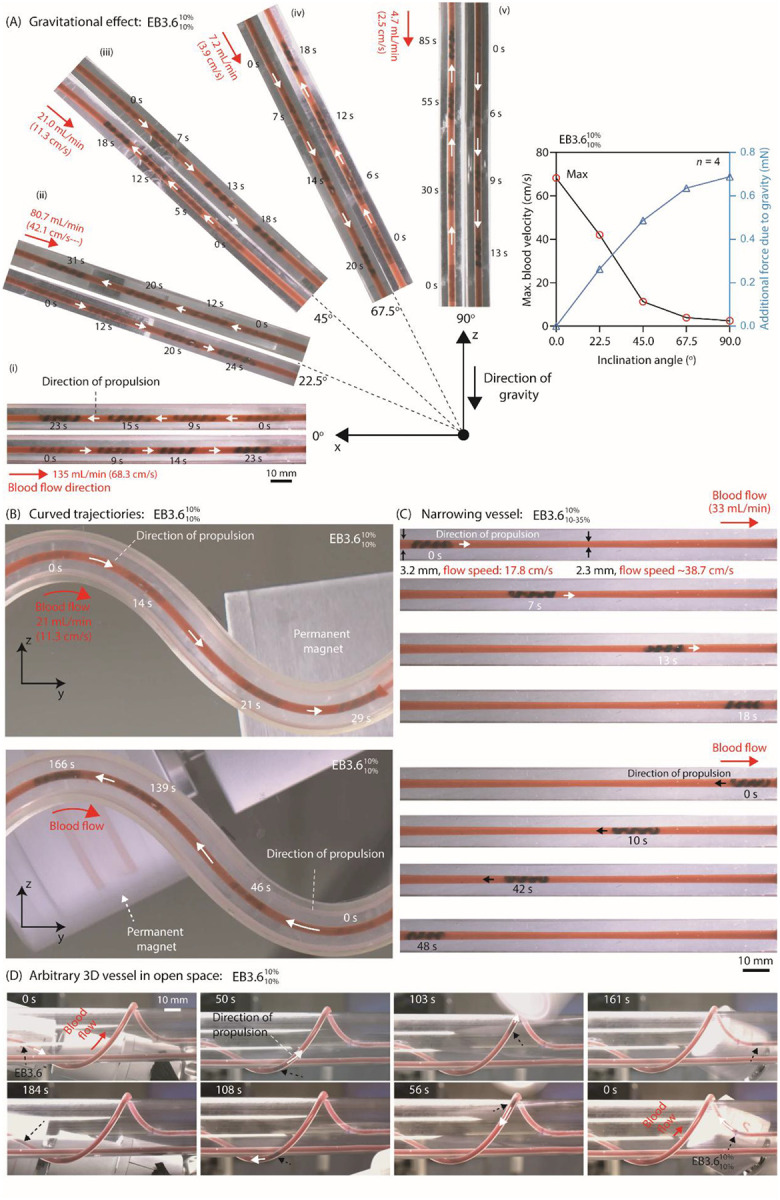
Magnetic propulsion performance of EB3.6 during multiphysical challenges. **(A)** Gravitational effect on ${\text{EB}3.6}_{10\%}^{10\%}$ propulsion assessed in a uniform phantom vessel by varying the inclination angle from 0° to 90°. For each inclination angle, the maximum blood-flow rate and velocity at which ${\text{EB}3.6}_{10\%}^{10\%}$ could move both with and against the blood flow was recorded. **(B)** Ability of ${\text{EB}3.6}_{10\%}^{10\%}$ to navigate curved trajectories evaluated along a sinusoidal phantom vessel. **(C)** Propulsion performance of ${\text{EB}3.6}_{10\%-35\%}^{10\%}$ through a nonuniform channel. **(D)** Navigation control of ${\text{EB}3.6}_{10\%}^{10\%}$ demonstrated in a human arm–sized, arbitrarily shaped 3D phantom vessel under various operating conditions of our permanent magnet: |B|=15-145mT, $f_{\text{m}}=30\;\text{Hz}$, $\left|v_{\text{m}}\right|=1-5\;\text{mm}/\text{s}$.

We further assessed the magnetic actuation performance under systematically controlled, multiphysical challenges including flow, gravity, vessel curvature, narrowing, and bifurcation. Tests in motion were conducted in various phantom vessels (Table S6) for both upstream and downstream round trips (Fig. 4A–D, Fig. S26, Supplementary movie 3). Based on these stability dynamics, we continued $\varepsilon_{\text{bias}}$ of 10% as the primary design configuration for determining EndoBot size relative to vessel diameter, as we conceive our short-term future applications focus on atraumatic immunomodulation at the venous endothelium and vessel walls.

We investigated the influence of gravity on propulsion in uniform vessel conditions by adjusting the inclination angle of straight-shaped phantom vessels from 0° to 90° (Fig. 4A). The best upstream motion performance was observed when the vessel was oriented perpendicular to gravity. In this vessel configuration, ${\text{EB}3.6}_{10\%}^{10\%}$ moved against a bloodstream flow rate up to 135 mL/min (68.3 cm/s). As the inclination angle increased, however, gravitational effects degraded the ability to control navigation against the blood flow. Despite the gravitational effects, ${\text{EB}3.6.}_{10\%}^{10\%}$ successfully climbed against gravity and navigated a bloodstream flow rate of 4.7 mL/min (2.5 cm/s) while maintaining precise magnetic control.

Next, we tested propulsion performance along a sinusoidal curve (Fig. 4B). ${\text{EB}3.6}_{10\%}^{10\%}$ successfully moved against a blood-flow rate of 21 mL/min (11.3 cm/s), performing comparably to its motion in a straight-line phantom vessel inclined at 45° to gravity (Fig. 4Aiii). Importantly, the robot’s bending through the curve did not negatively affect its locomotion behavior. We further evaluated propulsion performance through a narrowing channel (Fig. 4C). ${\text{EB}3.6}_{10\%-35\%}^{10\%}$ navigated blood flow at 33 mL/min, where the velocity reached 17.8 cm/s at a 3.2-mm diameter segment and 38.7 cm/s at a narrower 2.3-mm middle segment where the compressive strain reaches 35%.

Next, we evaluated navigation control of ${\text{EB}3.6}_{10\%}^{10\%}$ in an arbitrarily placed 3D phantom vessel, mimicking human brachial vein. It is wrapped around a human arm–sized open platform, creating a more challenging anatomical target than the typical extension of the brachial vein where the robot arm that controls the permanent magnet could move freely (Fig. 4D). $\text{EB}3.6_{10\%}^{10\%}$ could move against a maximum fluid flow of 7.2 mL/min (3.9 cm/s) seamlessly at any point of the vessel. The experimental results showed that under this 3D condition, the EndoBot performed similarly to its performance in the first test inclined at an approximate 67.5° angle. Finally, we tested navigation control of ${\text{EB}3.6}_{10\%}^{10\%}$ in a bifurcated Y-shaped vessel (Fig. S26). ${\text{EB}3.6}_{10\%}^{10\%}$ was able to propel both against and along the fluid flow at a maximum inlet rate of 21 mL/min (11.3 cm/s) with a bifurcation angle of 90°.

Across all motion tests, upstream navigation emerged as the limiting step. Notably, in all successful upstream motion conditions, ${\text{EB}3.6}_{10\%}^{10\%}$ could be seamlessly repositioned downstream without losing magnetic control, underscoring its robust capabilities for round-trip navigation.

### Wireless Deployment and Retrieval

Building on EndoBot’s stability in various flow conditions, magnetic navigation capabilities, and adaptive elasticity, we explored alternative wireless deployment and retrieval strategies applicable for different EndoBot sizes (Fig. S27, Supplementary movie 4). To demonstrate this approach, we used vascular access sheaths suitable for each EndoBot configuration. In one approach, EB3.6 was deployed using a custom-fabricated sheath with an inner diameter of 2.3 mm, whereas the other versions were deployed using Pinnacle introducer sheaths (Terumo): 7F for EB3.0 and EB2.5 (2.40-mm tip inner diameter) and 6F for EB2.5 (2.06-mm tip inner diameter). The sheaths were inserted into a phantom vessel with a blood-flow rate of 55 mL/min for the custom-fabricated sheath and 40 mL/min for the Pinnacle introducer sheaths.

Once the catheters were positioned within the vessel, we applied a rotating magnetic field $\left(|B|=90-145\;\text{mT},f_{\text{m}}=30\;\text{Hz}\right)$. As the EndoBots began rotating under magnetic actuation, the external magnet was moved at a speed ($\left|v_{\text{m}}\right|$) between 0 and 5 mm/s to guide the EndoBot out of the sheath and into the phantom vessel. Deployment was completed in under 15 seconds once the sheath reached the target-vessel segment. For retrieval, we experimented with various scenarios with varying degrees of success. In some but not all cases, EndoBot could move back upstream and be retrieved by the same catheter. The most reliable approach involved using a secondary catheter at the downstream end of the vessel segment. This catheter needed to be the same size or slightly larger (approximately 1F) than the initial deployment sheath. Our results showed that the EndoBot deployment and retrieval strategy is adaptable to various catheter sizes and compression configurations, offering flexibility for different vascular needs.

Collectively, these experiments demonstrate the EndoBot’s versatility and robust performance across a wide range of physiologic and anatomic conditions, suggesting it can operate effectively in animal and human blood vessels with similar dimensions and hemodynamic parameters (Table S1).

### Biocompatibility

#### Thrombogenicity

As a blood-contacting device, EndoBot and its corkscrew locomotion can have only minimal associated adverse interactions with blood and with ECs lining the vessel intima. Although the thrombogenicity of such devices is difficult to predict, we conducted a comprehensive series of tests to evaluate coagulative properties, hemolytic potential, platelet activation, and cytotoxicity of EndoBots. These tests followed standard in vitro protocols aligned with ISO-10993 Part 4 guidelines on biologic evaluations of medical devices^49^ and customized procedures tailored to our application context (Supplementary text 9)^50,51^.

#### Coagulation

**Figure 5.**
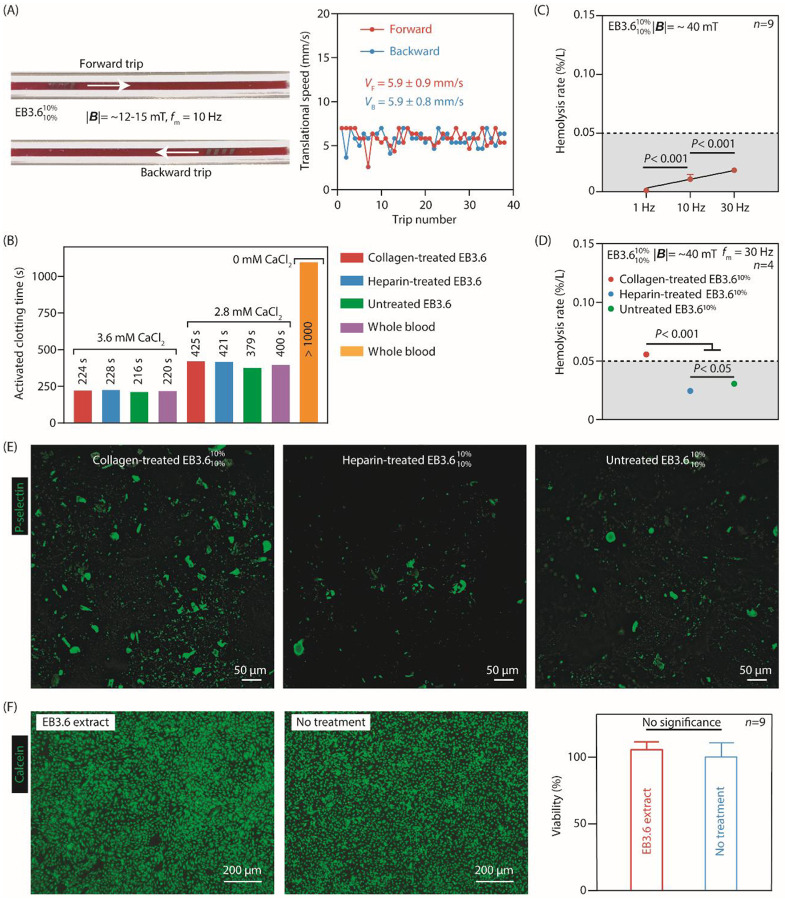
Biocompatibility of EndoBot in hemocompatibility (A-E) and cytotoxicity evaluations (F). **(A)** ${\text{EB}3.6}_{10\%}^{10\%}$ being continuously propelled forward and backward in a phantom vessel filled with fresh cow whole blood for approximately 40 round trips over 30 minutes, covering a total distance of about 560 cm. The consistent mean velocity throughout the experiment indicates that blood coagulation did not occur, which would have increased viscosity and drag. **(B)** Activated clotting time measured in single-donor human blood after it was exposed to various surface-treated EndoBots. **(C)** Hemolysis as a function of magnetic-field frequency, assessed in single-donor human blood and induced by the locomotion of $\text{EB}3.6_{10\%}^{10\%}$ across different magnetic field rotation frequencies. **(D)** Hemolysis resulting from the locomotion of ${\text{EB}3.6}_{10\%}^{10\%}$ after applying surface treatments. Collagen served as a positive control and heparin, a negative control, in single-donor human blood. **(E)** P-selectin–positive platelet binding and aggregation in response to EndoBot exposure. **(F)** Cytotoxicity of leachable materials from EndoBot on human umbilical vein endothelial cells, assessed using calcein AM staining and by counting relative number of live cells.

EndoBot operates for only a short time within a blood vessel (<15 min). During this period, its surface material composition and locomotion could potentially trigger blood coagulation. To assess this risk, we continuously monitored the real-time translational velocity of ${\text{EB}3.6}_{10\%}^{10\%}$ moving in a phantom vessel filled with whole bovine blood under constant magnetic actuation (approximately 12–15 mT, 10 Hz) for 30 minutes (Supplementary text 9, Fig. 5A, Fig. S28). Blood was preheparinized at 0.5 IU/mL, approximately matching the prophylactic dose recommended for adults undergoing endovascular procedures for deep vein thrombosis^52^. A decrease in the robot velocity over about 40 forward-backward traversals (approximately 560 cm total distance) would suggest coagulation-induced drag. However, mean velocities remained stable (5.9±0.9 mm/s forward and 5.9±0.8 mm/s backward), thus demonstrating no signs of coagulation-induced drag. This key observation provides translatable evidence for future animal experiments and human procedures.

We also measured the activated clotting time (ACT) of preheparinized, single-donor human blood after overnight contact with the device to further assess potential coagulation risks posed by the material composition of EndoBot. Heparin-treated and collagen-treated EndoBots served as negative and positive controls, respectively. Under these conditions, ACT values for the untreated EndoBot were comparable to both controls, indicating no heightened or reduced procoagulant effect (Supplementary text 9, Fig. 5B). This finding remained consistent even when calcium ion concentrations varied. Additionally, whole blood hemogram analyses revealed that all measured hematologic parameters remained within normal ranges, including hemoglobin, red blood cells (RBCs), hematocrit, platelet count, white blood cell count, and differential leukocyte counts (Supplementary text 9, Fig. S29), showing no adverse changes in blood cell morphology or counts. The absence of detectable procoagulant behavior also aligns with the designation of silica filler-free PDMS as a reference material for blood compatibility by the US National Heart, Lung, and Blood Institute^53^.

#### Hemolytic Effects

Hemolysis, RBC rupture, and subsequent release of intracellular components can be induced by mechanical stress or surface interactions. Such released molecules may, in turn, trigger thrombogenesis by activating platelets^54^. To evaluate potential hemolytic effects, we quantified free hemoglobin released from RBCs after exposing fresh, sodium citrate–treated bovine blood to ${\text{EB}3.6}_{10\%}^{10\%}$ in a straight-line phantom vessel at varying locomotion frequencies (1, 10, and 30 Hz) for 15 minutes (Supplementary text 9, Fig. S28). Higher magnetic actuation frequencies correlated with increased hemolysis, which we attribute to intensified mechanical shear forces at the blood-robot interface (Fig. 5C). Surface treatments also influenced hemolytic outcomes, with heparin-treated EndoBots producing slightly lower levels of hemolysis than untreated devices. Collagen-treated EndoBots had the highest levels of hemolysis (Fig. 5D). According to ASTM 756-17 (Standard Practice for the Assessment of Hemolytic Properties of Materials)^55^, materials are considered nonhemolytic if their hemolysis rate remains below 2% after blood contact^55^. Based on these criteria, EndoBot and its locomotion parameters had acceptable hemolytic behavior with lower than 0.1% hemolysis during all conditions investigated.

Subsequent to the hemolysis assessment, we examined blood-contacting inner surfaces of recovered EndoBots for platelet activation, adhesion, and aggregation, which are key indicators of thrombogenicity^51^. We used platelet P-selectin expression as a reliable marker to characterize platelet activation and aggregation^56^ (Supplementary text 9, Fig 5E). Consistent with the patterns of hemolysis, collagen-treated EndoBots had significantly elevated levels of P-selectin–expressing platelets attached to the robot surface, forming denser and larger aggregates. This outcome aligns with the known properties of collagen I, which presents epitopes for platelet binding and activation^57^. In contrast, heparin-treated EndoBots displayed substantially lower levels of P-selectin–positive platelet adhesion and aggregation. Although untreated EndoBots induced slightly more platelet activation and aggregation than heparin-treated EndoBots, these levels remained closer to those of the heparin-treated group, suggesting potential thrombogenic risk over long-term exposure.

#### Cytotoxicity

Tests were conducted to examine the effects of any leachable substances on human umbilical vein ECs (HUVECs) to evaluate the potential cytotoxicity of EndoBot. EndoBots were first incubated in EC culture medium for 1 week at 37° C. After incubation, the conditioned, undiluted medium was applied to HUVECs. The results showed no significant changes in cell viability or morphology for conditioned or untreated cultures (Supplementary text 9, Table S7, Fig. 5F).

These key findings demonstrate that EndoBot operates without inducing problematic coagulation, excessive hemolysis, substantial platelet activation, or cytotoxicity under the tested, short-term operational time frame and clinically relevant, preheparinized conditions. Its stable performance in both immobile and mobile conditions in blood flow, coupled with benign interactions with RBCs, platelets, and ECs, provides a promising foundation for safe, short-term endovascular applications. Future studies examining longer exposure times, varying heparinization levels and dosage patterns, and more physiologically complex models will further validate its biocompatibility.

### Fluoroscopic-guided Visualization, Localization, and Tracking In Vitro

Interventional fluoroscopy offers deep tissue penetration, high spatial resolution, and near real-time imaging, making it ideal for guiding small endovascular instruments, such as catheters. Although a few studies have demonstrated proof-of-concept X-ray-based control of milli/microrobots, direct visualization of these systems within the human body using a clinical fluoroscopic imaging platform has not yet been demonstrated, and the intrinsic challenges of achieving accurate 3D localization and tracking remain unaddressed^58,59^.

**Figure 6.**
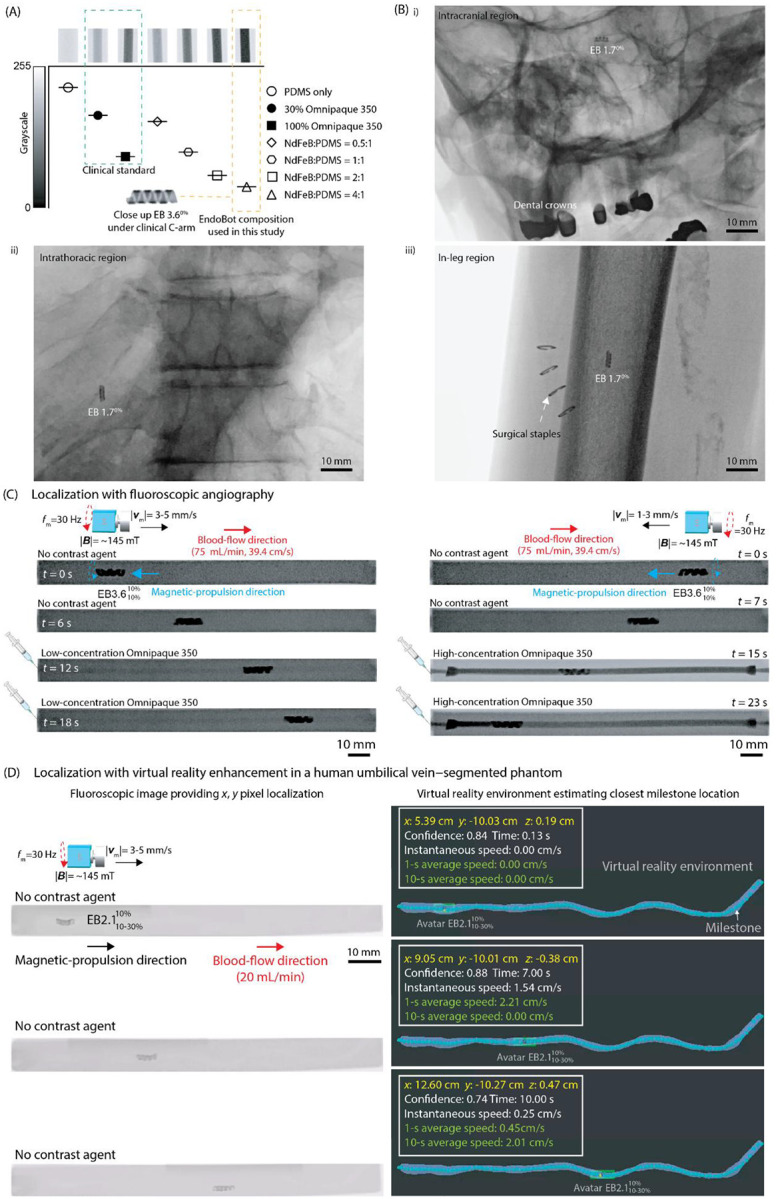
Challenges and clinically viable strategies for fluoroscopic-guided EndoBot navigation. **(A)** Fluoroscopic contrast optimization for EndoBot composites. Grayscale analysis of EndoBot precursor composites with varying neodymium-iron-boron:polydimethylsiloxane (NdFeB:PDMS) ratios (0.5:1 to 4:1 by weight) in 1-mL syringes, benchmarked against syringes filled with Omnipaque 350. A 30 vol % Omnipaque 350 solution defines the lower threshold for clinical visibility. **(B)** Representative fluoroscopic images demonstrating EndoBot visibility under clinically realistic attenuation. A EB1.7 remains clearly visible in human anatomical regions (i) intracranial, (ii) intrathoracic, and (iii) in-leg, despite attenuation from metallic artifacts such as dental crowns and surgical skin staples. (**C**, **D**) Fluoroscopic angiography-based localization. $\text{EB}3.6_{10\%}^{10\%}$ is visualized with and without contrast agent, is visualized with and without contrast agent, illustrating the limited depth perception and path identification capability of standard clinical fluoroscopy for safe, untethered robot control. **(D)** Digital-twin-guided localization and tracking. $\text{EB}2.1_{10\%-30\%}^{10\%}$ is localized within a segmented human-umbilical-vein-derived phantom without contrast injection, demonstrating the feasibility of an augmented, clinically viable framework for 3D navigation and control under routine fluoroscopic imaging

To enable direct integration of EndoBot within clinical interventional workflows, we first quantified its fluoroscopic visibility relative to the current medical imaging standard, Omnipaque 350 (GE Healthcare). Achieving sufficient contrast is critical for detecting the robot and monitoring its potential short- and long-term adverse effects as well as for managing patient radiation exposure^60^. To assess contrast, we filled 1-mL standard syringes with varying compositions of EndoBot precursor material, ie, different NdFeB:PDMS ratios, and compared their radiographic contrast to that of iohexol (Fig. 6A). For fluoroscopy-guided interventions, a medical device should exhibit contrast levels at least equivalent to 30% iohexol to ensure adequate visibility. Grayscale analysis of the samples revealed that all tested EndoBot precursor compositions exceeded the 30% iohexol threshold, indicating that EndoBot would be readily visible in a clinical setting. This superior radiographic visibility results from the higher atomic number of _60_Nd compared to _53_I, which increases x-ray attenuation and thus enhances detectability under a C-arm. Notably, the refined EndoBot composition (NdFeB:PDMS = 4:1) surpassed even the contrast level of 100% iohexol, outperforming this clinical standard. This analysis establishes the first quantitative framework for evaluating fluoroscopic detectability of untethered soft robots under clinical imaging conditions.

Next, we next validated EndoBot’s visibility in human anatomical regions relevant to potential interventional applications. EB1.7 remained clearly visible under fluoroscopy in (i) intracranial, (ii) intrathoracic, and (iii) in-leg regions of a cadaveric donor body despite attenuation from metallic artifacts such as dental crowns and superficial surgical staples (Fig. 6B, Figs. S30–32). Fluoroscopic visibility of EB1.7 in the in-arm and pelvic regions is also shown in Figs. S33–S34. These results demonstrate for the first time that untethered millirobots can be engineered for reliably visualization and localization under realistic clinical imaging conditions, establishing a foundation for safe image-guided navigation and control.

To demonstrate the magnetic propulsion of EndoBot under fluoroscopic guidance, we applied magnetic actuation within a phantom vessel at a high blood-flow rate of up to 75 mL/min (Fig. 6C, Supplementary movie 5). We continuously visualized EndoBot and its motion at 15 fps using cinefluoroscopic (cine) imaging mode and 8 fps using digital subtraction (DS) mode (Figs. 6C, S35, Supplementary movie 5). In DS mode, EndoBot remained stationary and was not initially visible due to the real-time frame subtraction process performed by the C-arm system. Once EndoBot started moving, it became clearly distinguishable against the blank background, leaving a white digital trace at its initial position. Although it is slower than cine mode, DS imaging has unique advantages for endovascular applications in the chest and head regions, where overlying bony structures can obscure vascular details.

Despite successful visualization of EndoBot and near real-time image acquisition during motion, both cine and DS fluoroscopy modes had substantial limitations. They provided limited structural information within the robot workspace. Without clear lumen visibility, localizing, tracking, and navigating the robot using external magnetic interactions could be severely compromised, endangering procedural safety. To address this, we performed fluoroscopic angiography, akin to established clinical procedures for guiding catheters and guidewires^61^. By injecting iohexol contrast into the bloodstream, we achieved lumen visualization proportional to the agent’s final blood concentration (Fig. 6C, Supplementary movie 5). Although fluoroscopic angiography is effective for localizing and tracking EndoBots and many other untethered millirobots and microrobots in vitro and ex vivo, adapting this method for in vivo use poses additional challenges. Unlike tethered devices, such as catheters, untethered devices like the EndoBot require continuous localization to prevent uncontrolled drift under magnetic propulsion. Periodic injections of contrast agents can aid localization; however, continuous administration is not feasible due to the nephrotoxicity of these agents and potential hypersensitivity reactions in patients^62^.

#### Digital Vascular Twin Mediated Localization and Tracking

To overcome these challenges, we recently developed an innovative virtual reality (VR)–based approach that integrates a digital twin of the robot’s operational environment, a robot avatar, and real-time robot position data^63^ (Supplementary text 10, Figs. S36, S37, S38). This framework synchronizes the physical and virtual workspaces through precise scale and orientation calibration, facilitated by positional data exchange through the Robot Operating System network. The robot avatar, a virtual representation of the physical robot, mirrors its real-time position and movements within the digital twin, enabling accurate and dynamic tracking in 3D space.

Fluoroscopic imaging inherently provides 2-dimensional (2D)-projection data, lacking z-axis information essential for 3D tracking. To overcome this limitation, we implemented an algorithm that infers z-axis positions by correlating 2D-positional data with the predefined geometry of a virtual model (Figs. S39, S40). This approach involves introducing virtual “milestones,” which are spherical markers with known 3D coordinates strategically placed manually along the central axis of the vessel (Figs. 6D, S41). These milestones, which span the entire navigable region within the vessel, provide a comprehensive spatial framework for localization. Each milestone is sized approximately one-quarter the length of EndoBot (about 3 mm), enabling precise segmentation of the robot’s trajectory. The segmentation framework allows the algorithm to infer z-axis positions directly from the 2D pixel data by mapping the robot’s projected location to the closest milestone in the virtual environment. By combining this inferred z-axis data with the x-y positional information from fluoroscopic imaging, we could construct accurate 3D-positional data sets (Figs. 6D, S41).

To demonstrate the feasibility of this approach, we created a digital twin of a human umbilical vein that was segmented from a fluoroscopic 3D scan (Supplementary text 10, Figs. 6D, S39-S41). The fluoroscopic stream did not detail any of the vascular trajectory as the model, ${\text{EB}2.1}_{10\%-30\%,}^{10\%}$ moved along the vessel. However, spatially calibrating the virtual and real environments allowed us to localize and track the motion of ${\text{EB}2.1}_{10\%-30\%}^{10\%}$ along the phantom vessel, with an average delay of approximately 66 ms in the digital twin (Fig. 6D, Supplementary text 10, Fig. S42).

Biplane fluoroscopy, which uses 2 x-ray sources, could address the limitation of 3D localization, but it also substantially increases overall radiation exposure^64^. Additionally, for magnetic robot control, biplane fluoroscopy imposes constraints on the external magnet and robotic arm, as x-ray beams are blocked in at least one plane at any given time. Expanding the virtual-twin concept offers a promising alternative, enabling precise 3D localization with a single x-ray source. Therefore, our VR-enhanced method substantially reduces reliance on contrast agents for localizing and tracking EndoBot while preserving the flexibility of robotic actuation in an open space. Additionally, the EndoBot detection algorithm consistently performed well, maintaining precise localization despite reduced visibility caused by the robot arm or magnet passing through the imaging field. Our previous results showed reliable tracking, even when the visual signal was nearly indistinguishable from background noise (*58*). Our future efforts will focus on extending the capability to 3D navigation in animal models and demonstrating its clinical viability.

### Atraumatic Fluoroscopic Navigation in Perfused Ex Vivo Human Umbilical Veins

**Figure 7.**
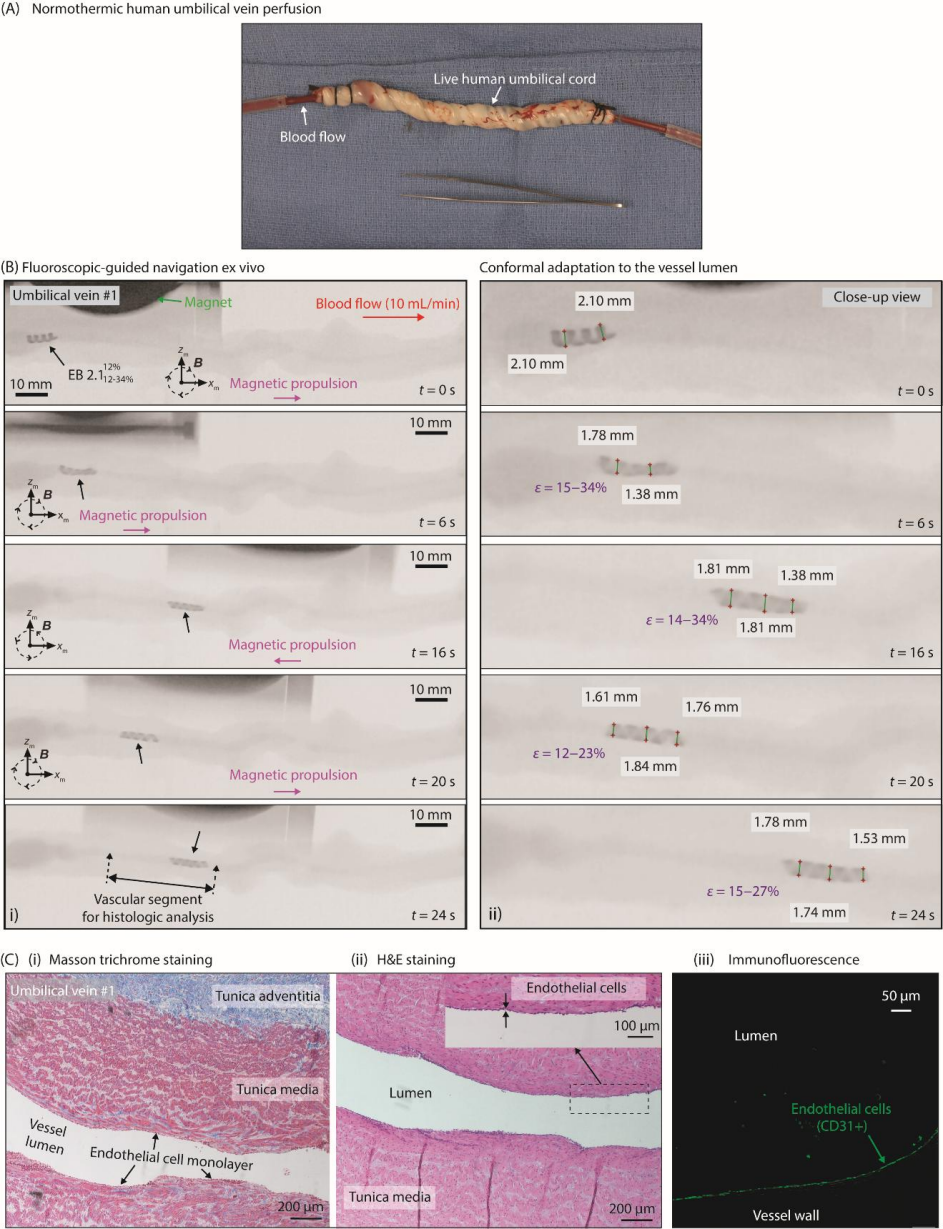
Fluoroscopic-guided EndoBot navigation in normothermically perfused, ex vivo human umbilical veins. **(A)** Reperfusion of a human umbilical vein with heparinized whole blood within one hour after cesarean section. **(B)** Fluoroscopic-guided navigation of ${\text{EB}2.1}_{12\%-34\%}^{15\%}$ in umbilical vein #1 using an iohexol contrast enhancer injected into the bloodstream. **(i)** EndoBot navigation in sequential snapshots. **(ii)** Zoomed-in view showing EndoBot’s diameter and conformal adaptation to the vessel at the corresponding time points. **(C)** Histologic analysis of longitudinal sections after four forward-and-backward passes along a 60-mm segment of umbilical vein #1 (as marked in B). **(i)** Masson trichrome. **(ii)** Hematoxylin-eosin. **(iii)** CD31/PECAM-1 (platelet endothelial-cell adhesion molecule-1). An intact endothelial-cell layer is shown with a preserved inner-vessel wall, demonstrating that robust EndoBot navigation took place without vascular injury under physiologic flow conditions.

We conducted controlled ex vivo navigation experiments using normothermically perfused (approximately 37 °C) human umbilical vein models to evaluate the mechanically adaptive, robust locomotion of EndoBot and its effect on vessel wall integrity. Fresh human umbilical cords were obtained immediately after cesarean sections, and reperfusion was established by circulating heparinized whole cow blood (200 IU/mL) through the umbilical vein using previously described protocols^65^ (Supplementary text 11, Figs. 7A, S43).

As with the phantom vessels, the perfused veins were invisible under the C-arm without contrast agents (Fig. S44). Therefore, Omnipaque 350 was injected into the closed circuitry of the bloodstream to enable uninterrupted visualization of the vessel throughout the experiment (Fig. S45). Based on the initial angiographic characterization of the minimum and maximum lumen diameters across the target segment, EB2.1 was chosen to ensure safe navigation by adhering to two fundamental design rules for EndoBot: Design Rule 1, maintaining continuous surface contact; and Design Rule 2, avoiding excessive deformation beyond allowable limits (Fig. 2A).

EndoBot moved forward and backward against and with a blood-flow rate of 10 mL/min in umbilical vein #1 (Figure 7Bi, Supplementary movie 6). Along the target vessel segment, ${\text{EndoBot}\;\text{EB}2.1}_{12\%-34\%}^{12\%}$ underwent dynamic elastic deformations ranging from approximately 12% to 34%, conforming to the irregular and likely vasoconstricted lumen caused by surgical trauma (Fig. 7Bii, moving in umbilical vein #1). Frame-by-frame analysis of the deformation patterns confirmed that EndoBot’s ability to elastically adapt to these irregularities enhanced the robustness and safety of navigation while maintaining blood-flow patency (Supplementary movie 6, Fig. 7B). This mechanically compliant design prevented excessive force on vessel walls, reducing the risk of localized damage to the endothelial lining.

In a worst-case scenario experiment using umbilical vein #2, EndoBot was subjected to supraphysiologic blood-flow conditions (50 mL/min, >200 cm/s flow velocity at regular segments) and extreme vessel dilation (Fig. S45). Even under these conditions, the magnetic stability of EndoBot was maintained in the dilated segment without drifting, successfully moving it into regular segments without losing its helical structure and disrupting the blood flow.

No evidence of EC denudation was observed after four round trips of EndoBot within a vein segment approximately 60-mm long (<5 minutes) during optimized actuation parameters $\left(|B|=\sim 145\;\text{mT},f_{\text{m}}=30\;\text{Hz}\right)$. Histologic analyses of the vascular segments navigated by EndoBot confirmed that the overall tissue morphology of umbilical veins #1 and #3 remained intact (Supplementary text 11, Figs. 7C, S46, S47). Hematoxylin-eosin and Masson trichrome staining showed preserved vessel-wall structure and the single-cell endothelial lining. Additionally, CD31/PECAM-1 staining validated the outer-lining cells to indeed be ECs, further confirming atraumatic navigation.

Although the umbilical veins each presented a distinct 3D vascular structure, EndoBot showed consistent and reproducible locomotion and safety outcomes across all tested models. For example, deformation for ${\text{EB}2.1}_{0\%-33\%}^{0\%}$ ranged from 0% to 33% in umbilical vein #2 (Figs. 7C, S45), whereas deformation for ${\text{EB}2.1}_{13\%-28\%}^{13\%}$ ranged from 13% to 28% in umbilical vein #3 (Fig. S47). These results demonstrate EndoBot’s capability to adapt safely to varying vessel geometries, supporting its potential applicability in diverse anatomic or pathological vasculature.

The ex vivo results emphasize the translational potential of EndoBot for safe and effective endovascular interventions. EndoBot’s mechanically adaptive locomotion ensures robust navigation in highly variable vessel geometries, such as stenoses, dilations, or vasospasms encountered in diseased vasculature. Furthermore, preserving vessel wall and EC integrity suggests that EndoBot could perform interventional tasks with less vascular trauma than traditional devices such as catheters or stents, which often cause endothelial denudation or mechanical injury.

### Endoluminal Drug Delivery

**Figure 8.**
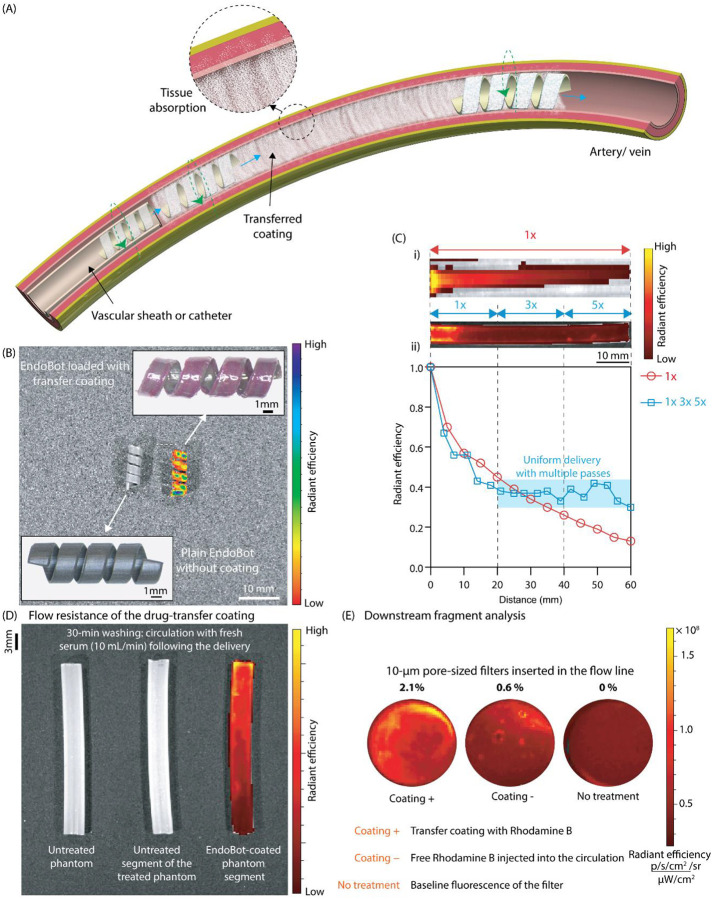
Endoluminal drug delivery using EndoBot. **(A)** Conceptual illustration. As EndoBot exits a vascular sheath or catheter and navigates through arteries or veins, its outer hydrophobic coating is gradually transferred to the vessel lumen by gentle mechanical rubbing against the vessel wall. **(B)** In vivo imaging system of an unloaded and loaded EndoBot with a refined transfer coating that incorporates Rhodamine B as a fluorescent reporter. This optimized formulation readily wets the surface of EndoBot, forming a stable, lubricious, and self-supporting outer layer. **(C) (i)** Endoluminal drug delivery $\left({\text{EB}3.6}_{10\%}^{10\%},|B|=50\;\text{mT},f_{\text{m}}=10\;\text{Hz}\right)$ along a 60-mm vascular phantom segment. **(ii)** Spatial distribution of Rhodamine B under continuous magnetic actuation modulated by adjusting the number of passes, thereby achieving precise spatial control and enhanced distribution uniformity along the vessel. **(D)** Coating stability against flow-induced erosion. The Rhodamine B–delivered vessel segment was recirculated with fresh serum at 10 mL/min for 30 minutes. The coating remained firmly attached to the vessel walls during the washing step. **(E)** Coating fragmentation analysis. After 30 minutes of closed-circuit circulation, only 2.1% of the circulating coating material was retained by the filter. Considering that free Rhodamine B exhibits approximately 0.6% nonspecific adsorption to the filter, about 98.5% of the coating material released into circulation consisted of fragments smaller than 10μm, confirming the safety of this drug-delivery procedure with minimal risk of downstream obstruction to perfusion.

As EndoBot moves through arteries or veins, it delivers its outer hydrophobic drug coating onto the lumen surface through gentle mechanical rubbing (Fig. 8A). Effective drug transfer relies on continuous surface crawling, ensuring stable contact between EndoBot and the vessel wall. This close interaction prevents the coating from being prematurely washed away by blood flow before it adheres securely to the vessel surface. For successful delivery, the coating must form a stable, flow-resistant drug depot that does not fragment into particles capable of obstructing smaller downstream vessels.

We developed this transfer coating using acetyl tributyl citrate (ATBC), an FDA-approved pharmaceutical excipient^66^. ATBC is a slightly hydrophobic plasticizer that prevents rapid drug dissolution into the bloodstream. Similar approaches have been employed for drug-coated balloon systems, such as the TransPax platform (Boston Scientific). TransPax combines a citrate ester excipient with low-dose paclitaxel ($2\;\mu \text{g}/{\text{mm}}^{2}$) for adhesion during balloon inflation^67^. ATBC has a blood half-life of approximately 0.5 hours in rats and 5 hours in humans, a result of serum esterase activity^66^. Moreover, it is rapidly metabolized by liver microsomes in both species (clearance half-life <30 minutes). These favorable properties make ATBC ideal for formulating biodegradable, blood-compatible carriers aimed at localized endoluminal drug delivery.

We incorporated poly(methyl methacrylate) (PMMA), another biocompatible hydrophobic polymer, to enhance the mechanical robustness of the coating, surface applicability, resistance to dissolution, and erosion stability (Supplementary text 12). Coating stability was evaluated using Rhodamine B/Fluorescein as a fluorescent reporter dye. Increasing the PMMA concentration resulted in coatings ranging from viscous to rigid, correspondingly improving their resistance to dissolution in bovine serum (Fig. S48A-C, Supplementary movie 7). We identified an optimal formulation containing 3.7 wt% PMMA in ATBC, achieving a balanced combination of ease of application onto the outer surface of EndoBot, enhanced aqueous dissolution resistance, and robustness against flow-induced erosion. To evaluate dissolution of the 3.7 wt% PMMA in ATBC coating and release of Fluorescein, we prepared thin coatings of systematically varied formulations at the bottom of glass vials and filled them with 20 mL bovine serum. Approximately 50% of Fluorescein release occurred within 3 hours and around 90% within 12 hours (Fig. S48C). The coating exhibited excellent stability in serum under harsh vortexing conditions for 5 minutes (Fig. S48D). Higher PMMA ratios yielded excessively rigid coatings unsuitable for transfer through mechanical rubbing.

The refined formulation readily wets EndoBot, creating a stable, lubricated, self-supporting outer layer (Fig. 8B). We assessed the potential impact of this thin coating on the mechanical properties of the entire construct by comparing the conformal shape adaptation of coated and uncoated EB3.6 configurations in a narrowing phantom vessel filled with whole blood (Fig. S49). The maximum deformation (approximately 42%) was comparable for both configurations with the same magnetic actuation, confirming the thin-transfer coating does not compromise mechanical safety, thus enabling its use with standard vascular sheaths in vitro and in vivo.

To showcase the endoluminal drug-delivery concept, we deployed ${\text{EB}3.6}_{10\%}^{10\%}$ along a 60-mm straight-line phantom vessel filled with bovine serum (Fig. 8C). Analysis from the in vivo imaging system (IVIS) revealed a gradual decrease in payload deposition along the segment after a single traversal (1x), which could be expected due to progressive coating depletion on the robot surface. Increasing traversal frequency (3x, 5x) produced a more uniform payload distribution, enabling spatial control and consistent coating efficiency along the vessel.

Next, we demonstrated the stability of the coating after deposit under flow-induced erosion by continuously circulating serum through a coated phantom vessel segment at 10 mL/min for 30 minutes (Fig. 8D). IVIS imaging confirmed that the coating remained on the vessel walls, indicating strong resistance to dissolution and shear stress. We did not detect nonspecific Rhodamine B deposition in regions of the phantom vessel without initial coating, further highlighting the success of the targeted delivery. The experiment showed that the coating forms a stable reservoir on the lumen surface that resists fast dissolution and shear forces. This stability potentially creates a time window for the drug to be absorbed locally across the vessel wall.

Fragmentation of the coating during and after delivery could affect downstream perfusion and lead to ischemic tissue injury. To evaluate this possibility, we placed a 10-μm pore-size filter in the circulation line. After 30 minutes of closed-circuit circulation of the EndoBot-mediated, coated phantom segment, only 2.1% of Rhodamine B–containing fragments within the circulation line were trapped in the filter (Fig. 8E). Free Rhodamine B exhibits about 0.6% nonspecific adsorption to the filter; therefore, about 98.5% of the fragments within the circulation were smaller than 10μm, confirming the safety of this delivery procedure with minimal potential risk to small-capillary perfusion.

This localized deposition method enables local, endoluminal pharmacotherapy previously unattainable with catheter-based systems.

### Fluoroscopic Navigation and Drug Delivery in Live Rat IVC

To evaluate the safety and performance of EndoBot for navigation and drug delivery in a live animal model, we selected the IVC of 300–375 g male Sprague-Dawley (SD) rats (N=5) as our testbed (Supplementary text 12). All animal procedures were conducted in accordance with the ethical guidelines and regulations established by the Institutional Animal Care and Use Committee of Mayo Clinic (IACUC, approval number A00007480-24) and National Institutes of Health guidelines.

The mean peak flow velocity in the IVC of an approximately 300 g male SD rat ranges from 6–10 cm/s^68^. We hypothesized that EndoBot would navigate safely in the rat IVC, given the successful performance of EB2.1 and EB3.6 in human umbilical veins (Fig. 7) and phantom vessels (Figs. 3, 4) at higher flow velocities in vessels with similar diameters.

#### Navigation

Based on intraoperative venograms, EB2.1 was selected as the optimal size for intervention, operated in a compression range of 5%-46% (Supplementary text 13, Table S8, Figs. S50-S56). As the IVC is a compliant vein with an elliptical and dynamically deformable lumen, its size varies with respiration and cardiac cycles, especially near the heart, where pulsatility is more pronounced. As a result, we realized that fluoroscopic estimation of lumen diameter carries inherent imprecision that needs to be accounted for in the device’s safety tolerance. Additionally, venography provides only transient visualization, since the contrast agent rapidly dissipates due to high venous return, limiting the time window for accurate anatomical assessment. This range corresponds to radial pressures below 0.75 kPa, which is within the safety thresholds derived from our observations of human umbilical veins (Fig. 7) and is consistent with previously reported limits for endothelial rupture (approximately 12.1 kPa)^6–10^.

**Figure 9.**
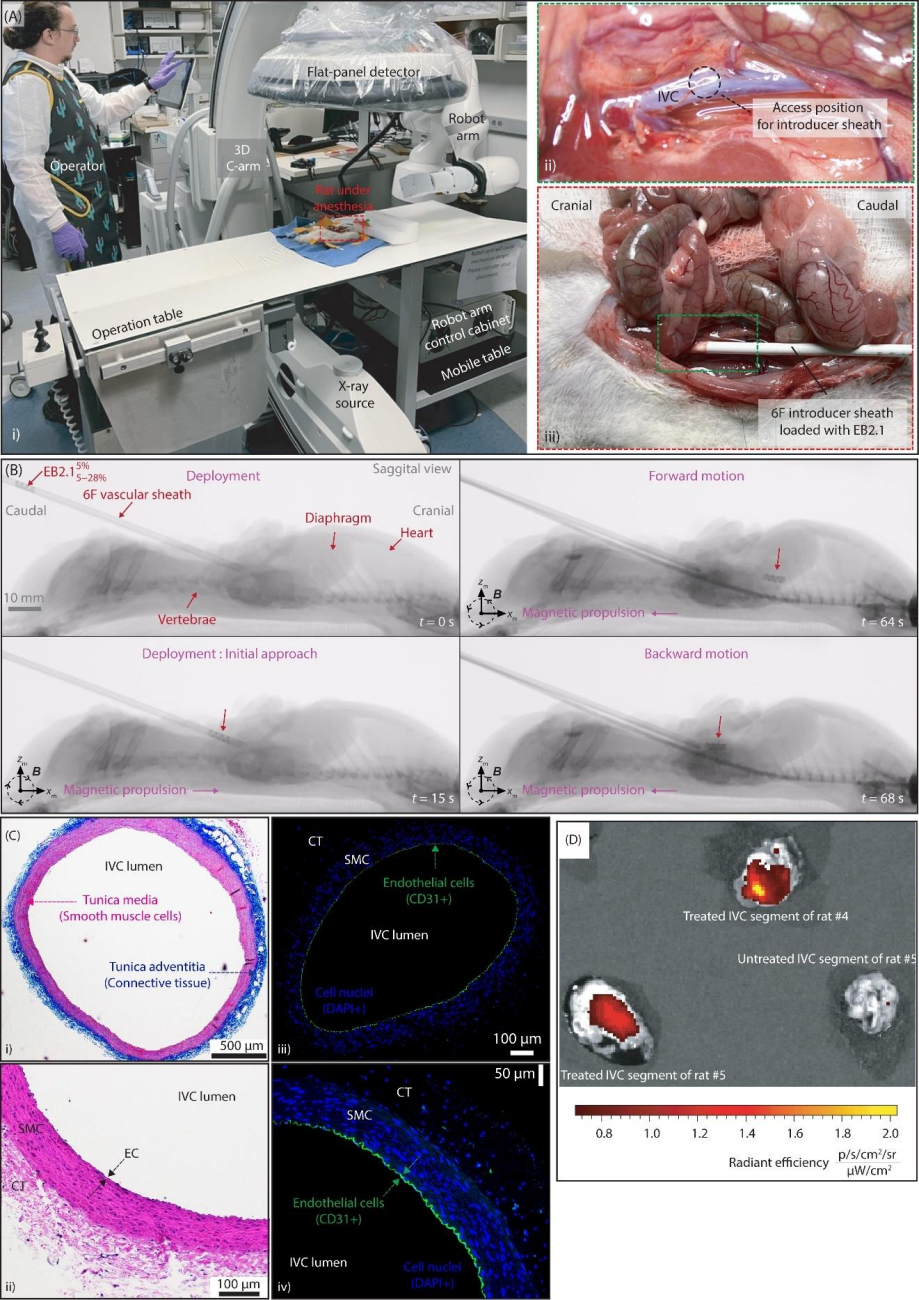
Fluoroscopic-guided navigation and drug delivery in the inferior vena cava (IVC) of live rats. **(A)** Experimental setup during EndoBot intervention: **(i)** Positioning of the vascular introducer sheath for IVC access. **(ii)** Close-up of the sheath insertion site in the anesthetized rat. **(iii)** Workspace configuration showing the human-scale, fluoroscopy-guided magnetic manipulation platform along with the anesthetized rat. **(B)** EndoBot navigation. Sequential snapshots depict the navigation of ${\text{EB}2.1}_{5\%-28\%}^{10\%}$ advancing and retreating along the IVC of rat #3. **(C) (i)** Masson trichrome, **(ii)** Hematoxylin-eosin (H&E), and (**iii**, **iv**) CD31 immunofluorescence marker–stained transverse sections of the IVC from rat #3 after five complete forward-and-backward passes, revealing an intact endothelial-cell layer and the preserved vessel wall, respectively. **(D)** In vivo imaging system examination of the extracted postmortem IVC, showing the successful delivery of Rhodamine B to a 30-mm segment of the vessel in rats #4 and #5, whereas no sign of Rhodamine B was observed in the nontreated segment from rat #5’s upstream IVC. CT indicates connective tissue; ECs, endothelial cells; IVC, inferior vena cava; SMC, smooth muscle cells.

The rats underwent general anesthesia, after which EB2.1 was deployed with 6F Pinnacle vascular sheaths using the Seldinger technique with minor adaptations (Supplementary text 13, Fig. S51). The EndoBot was magnetically actuated and positioned at the sheath tip. To prevent thrombosis within the tip, it was filled with 1,000 IU/mL of heparin dissolved in Omnipaque 350, which was injected (<0.5 mL) into the rat during intraoperative venography (Fig. 9A). EB2.1 was then deployed into the IVC and navigated along a 65-mm segment caudally, then back to the puncture site (Fig. 9B). We observed that the rat IVC exhibited forward-and-backward movements synchronized with the cardiac cycle, likely resulting from cardiac-induced motion of surrounding tissues (e.g., diaphragm, heart), rather than intrinsic venous pulsatility (Supplementary movies 8, 9). Despite these cardiac-induced vessel displacements, EB2.1 navigated the vessel effectively and maintained control, demonstrating its potential for navigating truly pulsatile vasculature in future applications. While the IVC, as a vein, is more compliant than a comparably sized artery, EndoBot exhibited even greater softness and adaptively conformed to the vessel diameter during navigation (Fig S52–56). EB2.1 successfully completed multiple forward-and-backward passes in rats #2 and #3 in under 5 minutes (2 and 5 passes, respectively). Throughout these passes, the total local radiation exposure from fluoroscopy remained below 20 mGy·cm^2^, which is within clinically acceptable safety thresholds (<200 mGy·cm^2^), ensuring minimal imaging-related risks^69^.

Immediately after EB2.1 navigation, the traversed IVC segments in rats #2 and #3 were isolated for histologic evaluation. No evidence of endothelial denudation, vessel wall disruption, or damage extending to the tunica media or adventitia was seen in the specimens (hematoxylin-eosin and Masson trichrome stains) (Fig. 9C). CD31+ immunofluorescence staining further confirmed endothelial cell layer remained intact, confirming the atraumatic nature and acute safety of EB2.1 navigation.

An additional important observation was made during the experiment with rat #3. During the initial venogram at device deployment, the fast contrast-agent injection caused the blood vessel to temporarily enlarge. As a result, EB2.1 rapidly slipped into the vessel and folded nonuniformly around the diaphragm narrowing, briefly losing its helical form. However, its preorganization into a helical structure, which endowed shape-memory properties, allowed it to regain its original helical structure with magnetic actuation. This accidental incident demonstrated a critical safety and reliability feature that will be valuable in clinical scenarios involving unexpected procedural complications.

#### Drug Delivery

To evaluate the in vivo performance and stability of the drug-transfer method, two EB2.1 devices were deployed in rats #4 and #5, each performing four forward-and-backward passes within the IVC. After navigation, normal blood flow was maintained for an additional 15 minutes before euthanasia to assess stability of the transfer coating. IVIS imaging of the harvested IVC segments showed successful and stable Rhodamine B delivery onto the luminal surface (Fig. 9D). The absence of fluorescence in a nontreated negative control segment from rat #5’s upstream IVC further confirmed the specificity and accuracy of the EndoBot-mediated drug-delivery method.

## DISCUSSION

EndoBot is designed to meet several critical performance criteria essential for safe and effective endoluminal drug delivery:

1. Robust locomotion: EndoBot can be steered reliably within a human-scale workspace inside 3D vessels under physiologic blood flow (Figs. 4, 6, 7, 9).
2. Mechanical adaptability: EndoBot’s soft structure conforms effectively to complex and variable vascular geometries, which is particularly important for safe interventions inside veins (Figs. 7, 9).
3. Stability: EndoBot maintains stable positioning within vessels, resisting drift under physiologic and supraphysiologic flow rates without disrupting normal blood flow (Fig. 3).
4. Fluoroscopic visibility: EndoBot delivers clinically compatible fluoroscopic imaging, enabling precise localization, real-time tracking, and navigational controls (Figs. 6, 7, 9).
5. Atraumatic tissue interactions: EndoBot avoids EC denudation and vessel wall injuries, ensuring atraumatic transit (Figs. 7, 9).
6. Biocompatibility: EndoBot has blood compatibility at heparinization levels comparable to those used in standard stent-placement procedures (Fig. 5).
7. Wireless deployment and retrieval: EndoBot can be deployed and retrieved using standard vascular access sheaths or catheters and magnetic propulsion (Fig. S27).
8. Controlled drug transfer: EndoBot provides uniform, tunable, flow-resistant deposition on vessel walls (Figs. 8, 9).

This study demonstrated the in vitro, ex vivo, and in vivo feasibility of each of these criteria and established a solid foundation for the translational development of EndoBot.

EndoBot presents a promising strategy to bridge a major gap in targeted endovascular delivery technologies by its ability to overcome the mechanical injury limitations inherent with current DCBs and DESs. Its ability to provide atraumatic, precise therapeutic delivery has the potential to shift current paradigms in vascular disease management, particularly through localized vascular immunomodulation for both primary and secondary prevention.

This platform holds broad translational potential across a range of occlusive vascular conditions, including bypass graft maintenance, prevention of restenosis after angioplasty, preventing stenosis in hemodialysis access lines, and post-stroke intervention.

Our specific next step goal is to establish a preventive intervention strategy that enables localized immunomodulation of AVF outflow veins in hemodialysis patients. Current AVF management strategies rely on repeated mechanical or pharmacologic interventions that can exacerbate endothelial injury and provoke inflammatory responses, contributing to restenosis and thrombosis^70^. The ability of EndoBot to perform atraumatic, localized, and image-guided drug delivery directly to the endothelium opens opportunities for preventive or immunomodulatory treatment of early intimal hyperplasia by inhibiting the infiltration of inflammatory monocytes and macrophages through endothelial cell contact. By initiating therapy at the time of surgical AVF creation, we aim to reduce local inflammation, inhibit intimal hyperplasia and venous stenosis, and ultimately extend the functional lifespan of venous access sites for patients with end-stage kidney disease (ESKD). Such applications exemplify the broader clinical potential of magnetically guided, physiologically compatible microrobotic systems for sustained vascular health maintenance and entirely non-invasive interventions.

The present system demonstrates feasibility within clinically relevant timeframes for localized vascular interventions involving small- to medium-sized vessels, such as AVF outflow veins, where the treated venous segment typically measures 10–30 cm in length. The KUKA LBR Med 7 R800 robotic arm provides a controlled magnetic workspace exceeding 40 cm, and the OEC 3D C-arm offers a 19 × 19 × 19 cm imaging field of view, fully encompassing the treatment zone without repositioning. These dimensions align with the anatomic range of localized vascular targets and are comparable to the configurations reported in other electromagnetic actuation studies^71^. Nevertheless, the current system has inherent limitations for anatomically complex or large-scale vascular territories, where continuous access without repositioning the C-arm or magnetic actuator is not yet feasible. Future work will focus on expanding the effective workspace, automating magnet positioning, and enhancing integration with clinical imaging systems. These developments will extend the applicability of EndoBot beyond small-scale vascular regions while maintaining its safety, precision, and clinical practicality.

In the long term, EndoBot’s mechanically adaptive architecture may enable non-invasive access to fragile or geometrically complex vascular regions that are currently unreachable with conventional tools, such as early- to mid-stage atherosclerotic plaques. Future studies will evaluate the safety and feasibility of EndoBot in such pathological contexts.

The customizable transfer-coating technology allows versatile delivery of diverse therapeutic agents, including small molecules, biomacromolecules, gene-editing vectors, and live cells. EndoBot’s adaptability extends beyond vascular indications to other luminal tissues, including the biliary tract, gastrointestinal tract, and urogenital tract, significantly broadening its impact within interventional medicine.

Although this study provided direct evidence that EndoBot does not cause immediate mechanical EC denudation, future research using survival animal models should evaluate its potential long-term effects on EC activation. The study also presents the feasibility of forming a stable drug depot on the blood vessel surface. Further survival animal experiments will focus on evaluating the absorption kinetics of pharmaceutical and gene-delivery agents to the endothelium and medial layer of the vessel wall.

We have identified several technical challenges that must be addressed for successful implementation of survival studies. Inadequate fluoroscopic visualization of the complex 3D vascular anatomy was a substantial obstacle encountered during the rat experiments, which hindered precise real-time localization, tracking, and navigation via external magnetic fields. Accurate navigation is essential for procedural success and for mitigating safety risks such as unintended device migration and potential vascular trauma. Although brief (< 1 s), intermittent injections of contrast agents improved vessel visibility, this strategy faced critical limitations: contrast agents rapidly diluted within the bloodstream, diffused into surrounding tissues, and failed to maintain a consistently visible lumen. This limited visualization resulted in loss of precise device control in the IVC of rat #1, causing accidental migration of EB2.1 into the heart (Supplementary movie 10). Such uncontrolled device migrations pose considerable safety risks, highlighting a critical need to improve medical image-guided localization, tracking, and real-time navigation capabilities.

Furthermore, the nephrotoxicity associated with contrast agents severely limits their frequent administration, especially in survival experiments or for patients with compromised kidney function, thereby compounding navigation challenges. To overcome these limitations, we propose leveraging the VR-enhanced EndoBot localization and tracking methodology outlined in Fig. 6. This strategy could substantially improve real-time visualization and navigation accuracy. Consequently, future research efforts will focus on refining this approach to enable precise 3D EndoBot navigation control utilizing single-source, 2D fluoroscopic imaging feedback.

## MATERIALS AND METHODS

The materials and methods required for understanding a specific result are integrated into the Results section. All other materials and methods are expanded within their respective texts in the Supplementary material.

## Supplementary Material

Supplementary Files

This is a list of supplementary files associated with this preprint. Click to download.

- 7SupMoviemov7.mp4
- 9SupMoviemov9.mp4
- 1SupMoviemov1.mp4
- 2SupMoviemov2.mp4
- 5SupMoviemov5.mp4
- 8SupMoviemov8.mp4
- 4SupMoviemov4.mp4
- 3SupMoviemov3.mp4
- 10SupMoviemov10.mp4
- 6SupMoviemov6.mp4
- SupplementaryMaterials.docx

## Data Availability

All raw and processed data are available from the senior author upon reasonable request.

## Abbreviations

2D: 2-dimensional
3D: 3-dimensional
ACT: activated clotting time
ATBC: acetyl tributyl citrate
AVFs: arteriovenous fistulas
*b*: groove
*b*: helical blade thickness
cine: cinefluoroscopic
DCBs: drug-coated balloons
*D*_bias_: biased diameter of EndoBot under bias compressive strain, ε_bias_
*D*_rec_: recovered diameter of EndoBot after unloading from a compressive strain, ε
*D*_max_: maximum luminal diameter
*D*_min_: minimum luminal diameter
*D*_r_: fabricated diameter
DESs: drug-eluting stents
DS: digital subtraction
*E*: elastic modulus
EB: EndoBot
ECs: endothelial cells
*ε*: strain
*ε*_bias_: bias compressive strain
eX-MMPt: X-ray–guided magnetic manipulation platform
FDA: US Food and Drug Administration
*λ*: pitch length
HUVECs: human umbilical vein endothelial cells
IVC: inferior vena cava
IVIS: in vivo imaging system
*L*: length
NdFeB: neodymium, iron, and boron
PDMS: polydimethylsiloxane
*φ*,: helical angle
PMMA: poly(methyl methacrylate)
RBCs: red blood cells
SD: Sprague-Dawley
*T*: thickness
VR: virtual reality
